# Are fire regimes the result of top-down or bottom-up drivers?

**DOI:** 10.1098/rstb.2023.0447

**Published:** 2025-04-17

**Authors:** Juli G. Pausas, Jon E. Keeley, Alexandra D. Syphard

**Affiliations:** ^1^ Desertification Research Center (CIDE), CSIC-UV-GV, Moncada, Valencia 46113, Spain; ^2^ US Geological Survey, Western Ecological Research Center, Three Rivers, CA 93271, USA; ^3^ University of California-Los Angeles, Los Angeles, CA 90095, USA; ^4^ Conservation Biology Institute, Corvallis, OR 97333, USA

**Keywords:** fire regime, boreal forest, surface fires, crown fires, *Pinus*, *Picea*, Pinaceae, Eurasia, North America

## Abstract

The evolutionary topic we examine here is whether species determine the environment (bottom-up) or if environments shape plant traits (top-down). For the environment, we focus on the fire regime. Many forests are subject to either frequent low-intensity surface fires or less frequent but high-intensity crown fires. What are the ultimate factors controlling these fire regimes? The top-down model proposes that environmental factors controlling productivity and ignitions shape fire regimes; the bottom-up model attributes them to different plant assemblies. In boreal forests, it is assumed that, because of the similar climate, forests of North America and Eurasia undergo distinct fire regimes (crown-fire and surface-fire regimes, respectively) due to bottom-up forces. We tested the hypothesis that fire regimes are primarily controlled by top-down factors by selecting congeneric species of *Pinus* and *Picea* from both continents. Plots dominated by each species were studied using remote sensing data. We then compared environmental conditions where the species occur and found that Eurasian tree species occur in warmer and more productive environments than North American tree species. Our results support the top-down model, which suggests that environmental factors control the surface- versus crown-fire regime in boreal forests.

This article is part of the theme issue ‘Novel fire regimes under climate changes and human influences: impacts, ecosystem responses and feedbacks’.

## 1. Introduction

Two evolutionary mechanisms have been proposed that drive the relation between organisms and their environment. One is that the environment selects for species with appropriate traits (top-down [1,2]). Another is that species generate the appropriate environment for their persistence (bottom-up [3,4]). These two mechanisms are also important to understanding drivers that ultimately shape fire regimes (i.e. the characteristic fire activity that prevails in a given area). Despite fire regimes varying considerably depending on their different components (e.g. frequency, intensity, seasonality), broadly speaking there are two fire regime types: periodic high-intensity crown fires and frequent low-intensity surface fires. What is the ultimate factor determining the predominant type of fire regime in forest ecosystems? The proximal cause (i.e. the phenomenon that is immediately responsible or the ‘how’ explanation) is whether the surface fire is intense enough to transition into the crown (e.g. [5,6]); however, here we ask about the factors that control the intensity of fires (e.g. factors that allow the development of ladder fuels and the change in forest structure), which is the ultimate or distal cause (the ‘why’ explanation).

In explaining the distribution of different fire adaptations in the genus *Pinus,* it was proposed that ultimately fire regimes were driven by the environment (top-down approach), specifically the combination of site productivity and frequency of ignitions [7]. Moderately high-productivity forests allow trees to outgrow surface fires, and with frequent ignitions, surface fuels do not accumulate, thereby selecting for a particular suite of plant traits. These traits include high tree growth rates and self-pruning, which prevent the spread of fires from surface fuels to crowns, and a thick basal bark, which protects from the heat of surface fires. In contrast, low-productivity environments with infrequent fires produce fuel loads conducive to a crown-fire regime. This is linked to a suite of traits such as branch retention, which enhances flammability, and serotiny, which allows reproduction after crown fires [7].

**Figure 1.**
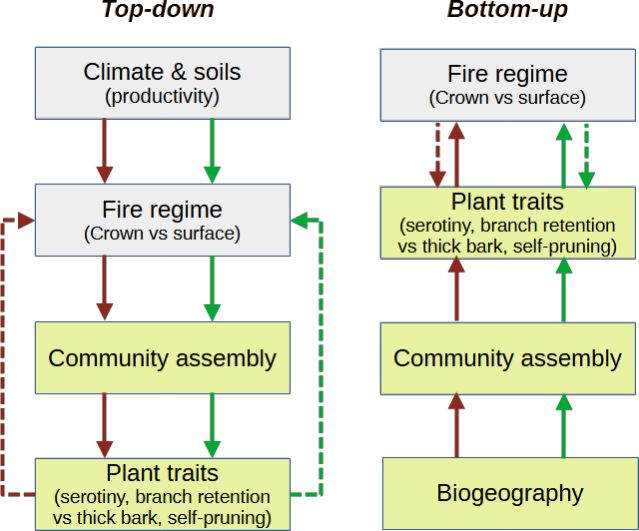
Simplified scheme of the two evolutionary mechanisms explaining the relationship between plants (light green boxes) and their environment (grey boxes). The top-down mechanism (left) suggests that there is a variability in environmental factors (e.g. productivity in different continents) that generates different fire regimes, which assemble different communities with different plant traits. The bottom-up mechanism (right) suggests that the different biogeographical history (in a given environment, e.g. boreal climate) assembles different communities with different plant traits that subsequently generate different fire regimes. Flammability traits (e.g. branch retention versus self-pruning) may feed back to the fire regime (dashed lines), enhancing the difference between crown and surface fire ecosystems. For plant traits, serotiny and branch retention are examples of traits associated with crown fires, while thick basal bark and self-pruning are associated with surface fires. Red and green arrows are different pathways (e.g. different productivity, fire regime, community and traits) related to crown and surface fire regimes.

More recently, a bottom-up model has been proposed to explain the ultimate factor determining the predominant type of fire regime in boreal conifer forests [8]. This hypothesis was based on observations suggesting that ‘the’ climate across Eurasian boreal forests was similar to that in North American boreal forests. Since the regions are characterized by very different fire regimes (surface versus crown, respectively [9–11]), those authors concluded that the fire regime was not driven by top-down factors that mediated plant traits, but rather by bottom-up characteristics of the available species pool in each biogeographical region. That is, whereas the top-down model suggests that the environmental difference (productivity) is the key factor determining the fire regime type and plant traits, the bottom-up model attributes the fire regime to differences in community assembly, which in turn is driven by the different biogeographical history (figure 1). For the bottom-up model to hold, the key requirement is that growing conditions are the same—and thus species’ differences must dictate differences in fire regime. Given this key assumption, we can investigate the fundamental controls over boreal fire regimes by comparing growing conditions in the two continents.

Our hypothesis is that the broad-scale variability of forest fire regimes (crown versus surface fires) is primarily controlled by top-down (environmental) factors. To test this hypothesis, we selected tree species pairs in *Pinus* and *Picea* that occur in the boreal forest of Eurasia (dominated by surface fire regimes [9,11–13]) and America (dominated by crown fire regimes [8,10]) and examined the environmental parameters where they occur. Specifically, we chose North American *Pinus* and *Picea* species that do not self-prune and have serotinous cones typical of crown-fire regimes; Eurasian species in these two genera lack serotiny and typically experience surface fire regimes. In fact, there are no serotinous trees in the Eurasian boreal forest. We predict that the environmental variability will be greater between continents (within the same genus) than within the continent (for species of the two genera). More specifically, we predict that Eurasian boreal species grow in more productive environments conducive to surface fire regimes, whereas North American boreal species are in less productive environments conducive to crown fire regimes. If this is the case, it would support the hypothesis that forest fire regimes are driven by top-down factors rather than by bottom-up factors.

## 2. Methods

### (a) Species

We selected two pairs of coniferous species that are abundant in the boreal forest: *Picea mariana* and *Pinus banksiana* in North America, and the congeneric *Picea abies* (northern lineage [14]) and *Pinus sylvestris* in Eurasia. These species were selected because they are common and widespread in the boreal forest, occur under contrasting fire regimes, and have different fire-related traits; in addition, they allow congeneric comparisons. Specifically, the two North American species are serotinous and typically retain lower branches, which contribute to crown fires, whereas this is not true for the two Eurasian species, which commonly burn in surface fires. Of the two Eurasian species, *P. sylvestris* is a typical surface-fire regime species [12,13] with self-pruning that prevents the continuity of vertical fuels. *Picea abies* at younger ages does not self-prune lower branches, but its low flammability allows this species to withstand some surface fires; older trees do prune lower branches [13]. The species selected allowed us to compare closely related (within genus) taxa between continents (congeneric comparison). This means that many plant traits are similar owing to recent common ancestry, and thus it is a way to better isolate the question (climate and fire regimes) from other factors.

We extracted localities (occurrence coordinates) for the four species from the Global Biodiversity Information Facility (GBIF; for discussion on the accuracy of this dataset see e.g. [15,16]) within areas defined as boreal forests from a global map of terrestrial ecoregions [17]. Using the package ‘rgbif’ [18] in the R statistical program [19], we obtained a total of 13 479 GBIF records for the four species. We then cleaned records with duplicate coordinates and excluded those plots that were outside the species distribution area (e.g. obvious spatial errors or located within human-dominated land covers). Finally, to minimize the potential for spatial autocorrelation, we filtered the records using a minimum separation distance of 1 km [15]. This yielded 8012 plots for the four species distributed as follows: *P. abies* (*n* = 4074), *P. mariana* (*n* = 881), *P. sylvestris* (*n* = 2488) and *P. banksiana* (*n* = 569).

### (b) Fire regime

It is difficult to quantify the difference in fire regime (surface versus crown) between continents. One approach is to evaluate the differences in fire intensity from remotely sensed data, under the expectation of higher fire intensity in the North American boreal forest where crown fires are common [8,10,11]. To do so, we extracted the MODIS hotspots (Collection 6 Active Fire Products from Terra satellite, dataset MCD14ML; downloaded from the University of Maryland, USA; period 2002–2022) for a pixel of 0.1° around each plot (8012 locations). We then averaged the fire radiative power (FRP, in MW, an indicator of fire intensity) for those plots with hotspots during that period. This method had limitations (e.g. some understorey fires remain undetected, a short time window, and FRP may not only reflect fire intensity) but may still be an indicator of the difference in fire regime in our selected plots.

### (c) Environmental information

For each of the 8012 locations, we extracted environmental data from global datasets as follows. We used Google Earth Engine [20] to extract net primary productivity and soil data. Net primary productivity (kgC m^−2^ yr^−1^) was obtained from MOD17A3HGF.061 (Terra Net Primary Production Gap-Filled Yearly Global 500 m, https://lpdaac.usgs.gov/products/mod17a3hgfv061/) and soil properties from the SoilGrids dataset (https://www.isric.org/explore/soilgrids; 250 m resolution, ISRIC World Soil Information). We selected the bottom soil layer as it best reflects the parent material (lithology); top soil layers may be influenced by the vegetation. For climate variables, we used bioclimatic data from [21], mapped at a resolution of 30 arcsec (i.e. about 0.46 km at 60° latitude).

### (d) Statistical analyses

For each environmental variable, we estimated the variance component, using mixed-effects models where species was a random factor nested within continent. This allowed us to evaluate the amount of environmental variability distributed between continents (i.e. within congeneric species), within continents (i.e. between genera) or with species (i.e. the spatial variability across the species distribution). We used the ‘nlme’ library in R [22].

For each environmental variable, we evaluated the differences between species from different continents (North America versus Eurasia) within each genus (*Picea* and *Pinus*) by looking at the frequency distribution of each variable. We then compared the means among continents (between species of the same genus) using a maximum likelihood estimation of spatial simultaneous autoregressive (SAR) lag; this method explicitly accounted for the spatial dependence between observations (using geographical coordinates of the plots). The SAR models were performed using the R library ‘spatialreg’ [23].

Unless otherwise stated, figures are displayed for the entire dataset (*n* = 8012), but statistical analyses (variance component, SAR) were performed with 500 randomly selected plots for each species (*n* = 2000). Different random selections provide very similar results, and thus they are not considered further. When using GBIF data, subsetting for the statistics further reduces the spatial autocorrelation and produces better predictive models [15].

## 3. Results

**Figure 2.**
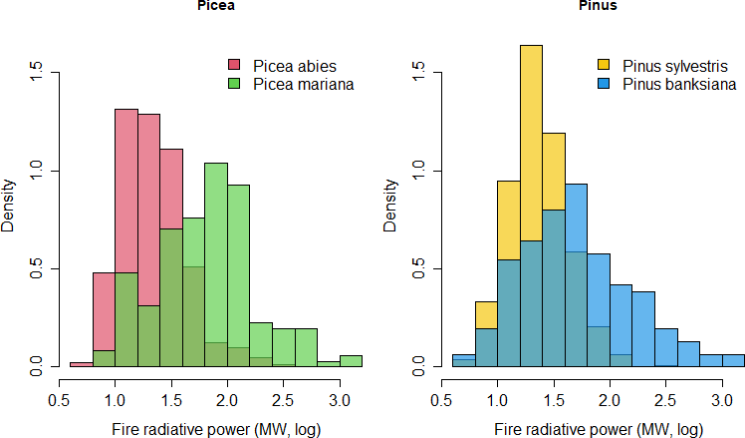
Mean fire radiative power for each plot (log-scale), an indicator of fire intensity as detected from MODIS remote sensors. The figures show the comparison between continents for *Picea* (*P. abies* in Eurasia and *P. mariana* in North America) and *Pinus* (*P. sylvestris* in Eurasia and *P. banksiana* in North America). Note that the darker green (in *Picea*) and dark blue (in *Pinus*) are overlapping zones of the frequency distribution between the species.

Remotely sensed hotspots varied in FRP between continents, but not between species of the same continent (table 1); they also vary greatly within each species. The frequency distribution suggests that, for the species considered, fires in the Eurasian forests have lower FRP than North American species (figure 2); the differences between continents were significant (table 2).

**Table 1.** Variance component estimates (scaled to 0–1) for environmental variables between continents (within genus), between species of the same continent (i.e. between genera) and within species. The last column (figure) indicates the corresponding figure where the frequency distribution is displayed. Values are estimated from 500 randomly selected plots for each of the four species. For each variable (i.e. by rows), the highest values are in bold and the lowest in italics.

variable	between continents	between species (same continent)	within species	figure
fire radiative power	0.156	*0.001*	**0.844**	2
net primary productivity	**0.476**	*0.110*	0.416	3
soil pH	**0.536**	*0.072*	0.392	3
total soil nitrogen	0.310	*0.001*	**0.685**	electronic supplementary material, figure S1
mean annual temperature	0.449	*0.021*	**0.529**	electronic supplementary material, figure S1
minimum temperature (coldest month)	**0.653**	*0.187*	0.328	3
annual temperature range	**0.606**	0.048	*0.346*	electronic supplementary material, figure S1
annual precipitation	*0.000*	0.018	**0.983**	electronic supplementary material, figure S1
precipitation seasonality	0.090	*0.082*	**0.828**	electronic supplementary material, figure S1

**Table 2.** Results of the comparisons of the means between continents for each variable and genus, using spatial simultaneous autoregressive (SAR) lag models. Shown are the *p*-value of the comparison, the SAR coefficient (rho) and the Lagrange multiplier test for residual autocorrelation. The data for these comparisons are displayed in figure 3 and electronic supplementary material, figure S1. In all cases, the comparison between a linear model and this SAR model is significant (Likelihood-ratio test < 0.001), suggesting that including the spatial component improved the model. ****p < 0.0001; n.s., *p* > 0.05.

	*Picea*			*Pinus*		
variable	model	rho	residuals	model	rho	residuals
fire radiative power	****	0.21	n.s.	****	0.26	n.s.
net primary productivity	****	0.61	****	****	0.60	0.0001
soil pH	****	0.70	0.012	****	0.77	****
total soil nitrogen	****	0.73	n.s.	****	0.74	****
mean annual temperature	****	0.86	n.s.	****	0.85	n.s.
minimum temperature (coldest month)	****	0.88	0.008	****	0.89	n.s.
annual temperature range	****	0.82	0.002	****	0.85	****
annual precipitation	n.s.	0.73	n.s.	0.028	0.81	n.s.
precipitation seasonality	****	0.88	n.s.	0.008	0.91	0.047

**Figure 3.**
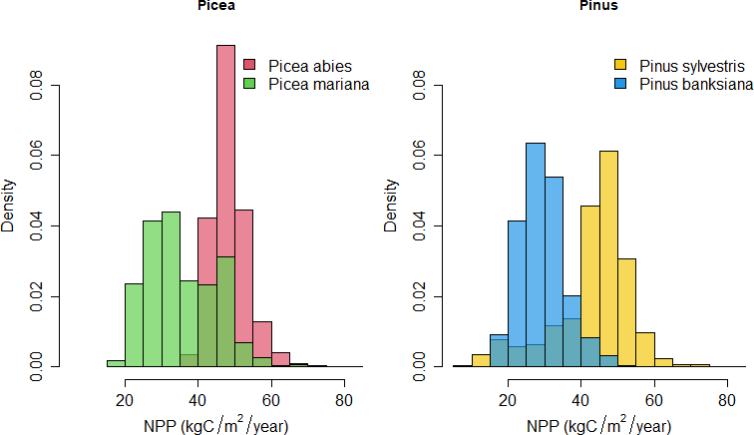
Frequency distributions of environmental variables for each species. The figures show the comparison between continents for *Picea* (*P. abies* in Eurasia and *P. mariana* in North America) and *Pinus* (*P. sylvestris* in Eurasia and *P. banksiana* in North America). NPP, net primary productivity. The difference between congeneric species (i.e. between continents) is shown in table 2. For more environmental variables, see electronic supplementary material, figure S1.

Primary productivity, climatic variables related to temperature, and soil pH showed the strongest variability between continents, while the variability between species within the same continent was low (table 1). There was also considerable variability within species as those species covered large areas in the boreal region. Precipitation-related variables varied substantially across the species’ distributions, with little remaining variability between continents. The variability between species co-occurring in the same continent was very low for all variables (table 1). Thus, the environmental conditions where the species occurred were more different between continents (within genus) than within continents (between genus).

Eurasian *Picea* and *Pinus* species grow in higher productivity sites under warmer conditions and with a lower annual temperature range than North American ones (figure 3; electronic supplementary material, figure S1). They also occur on sites with lower soil pH (figure 3). Thus, the environmental niches of the congeneric species living in different continents are different (electronic supplementary material, figure S2). Mean differences between continents, when considering the spatial structure of the plots, are significant for all variables except for annual precipitation (table 2). All environmental variables showed a high and positive spatial autoregressive coefficient (rho > 0.59; table 2). Some spatial autocorrelation remained in the residuals for some of the variables (table 2).

## 4. Discussion

North American boreal forests show more intense fires than Eurasian boreal forests, in agreement with their different fire regimes (crown versus surface fires, respectively [8]). These different fire regimes within a given biome (the boreal forest) have puzzled ecologists given their presumed similarity in environmental conditions [8,10,11,24]. The regional similarity in climates has led to the proposal that the differences in fire regimes are due to a bottom-up mechanism—such that species with different traits (from different biogeographical pools) generate different fire regimes [8]. The results of our investigation, however, show some environmental differences between the continents, thereby supporting an alternative hypothesis that the broad-brush approach of comparing continental climates has confounding effects when addressing fire regimes. At a broad scale, these regions may have similar climates [8]; however, there is substantial variation when considering the localized environmental factors that characterize the species distributions. That is, some widespread species in the American boreal forest inhabit environments that are significantly different from those in common species in Eurasia, despite the overall climate similarity between continents.

Our results demonstrate that congeneric pairs of *Pinus* and *Picea* species with different character trait syndromes and subject to different fire regimes between American and Eurasian boreal forests occupy significantly different environments on the two continents. Specifically, boreal Eurasian species occur in warmer and more productive environments with a lower temperature range throughout the year than the North American boreal trees. These more productive environments provide better growing conditions for conifers to grow faster and taller, thus limiting the ability of surface fires to spread to their crowns (*P. sylvestris*; fire tolerator syndrome [13,25,26]), or to quickly develop a low-flammability structure, especially in wetter microsites (*P. abies*; fire avoider [27]). The self-pruning in *P. sylvestris* further limits surface fire from reaching the crowns. The current higher incidence of lightning flashes in Eurasia [8], if consistent throughout the past, may have also contributed to a high fire frequency, which in turn maintained low fuel levels and surface fires. In contrast, North American conifers are highly flammable and grow in less productive environments; thus, they typically lack a gap in vegetation between surface and crown fuels; so fires easily reach the crown. In such conditions, conifers have evolved serotinous cones to successfully reproduce under crown-fire regimes (fire embracer or post-fire seeder syndrome). On the other hand, no serotinous species are known to occur in the Eurasian boreal forests. The two fire regimes are maintained by feedback processes such as those driven by flammability-related traits (dashed lines in figure 1 [28,29]). That is, the high flammability of trees of North American species (branch retention; high proneness to crown-fire) contrasts with the low flammability of Eurasian species (self-pruning; low proneness to crown-fire), and this contrast enhances the divergence between North American and Eurasian fire regimes. This dynamic interplay is evident at the ecological scale (surface fires consume lower branches and enhance the vertical gap) and the evolutionary scale (driving divergent trait selection between fire regimes) and stabilizes the system into two contrasting fire regimes. Once the two systems are established, those feedbacks could potentially maintain the two fire regimes in similar environments, as suggested by some niche overlap (figures 1 and 2).

Overall, the top-down explanation of the contrasting fire regimes is more parsimonious than the bottom-up explanation. That is, the ultimate cause of divergent fire regimes between continents is the difference in environmental conditions that generate different evolutionary dynamics rather than the difference in assemblies due to biogeographical history (figure 1). That is, the subcontinental variability in top-down factors is a key to understanding the different fire regimes and associated plant traits between continents. The proximal cause (the ‘how’ explanation) is obviously driven by the accumulation of flammable fuel ladders in the North American boreal forests [6], as has been reported in many other coniferous forests [30,31]. We further contend that, at a broad scale, fire regimes are largely driven by environmental factors, which control both site productivity and the frequency of ignitions (e.g. [7,32,33]).

The fact that variability is higher between congeneric species (from different continents) than between coexisting species from different genera suggests that, in boreal forests, the fire regime is a strong evolutionary force which makes the ancestral niche evolutionarily labile (low niche conservatism; allopatric speciation). This is despite the general tendency in plants for niche conservatism [34] and supports the idea of evolutionary feedbacks between fire regimes and plant traits. In fact, our results suggest that fire regimes have generated a convergence in niche and fire traits (e.g. serotiny) between different genera. This is similar to what we observed in Mediterranean-climate regions (MCRs), where there are many cases of convergent evolution of fire traits in different lineages [35]. While some phylogenetic constraints may generate differences (especially when comparing northern and southern MCRs), the influence of top-down processes is strong enough to produce similar fire regimes and many instances of convergent evolution in MCRs.

An important consideration in determining variation in fire regimes, as in any other ecological process, is the scale of analysis [36]. Our investigation, and the example of MCRs above, focused on broad-scale controls of contrasting fire regimes that are maintained at evolutionary scales and suggest the importance of top-down factors. However, bottom-up factors can be influential at smaller scales where two fire regimes may coexist in the same climate (i.e. within the same species pool). For instance, in forest–savannah landscape mosaics, shade produced by forest trees is associated with high moisture and low wind and favours low fire activity in forests, while in neighbouring savannahs, the higher light and wind generate drier conditions that maintain frequent fires and flammable grasslands [33,37]. The two co-occurring biomes (forest and savannahs) have plants with markedly different traits. That is, at small spatial scales, vegetation feedbacks can drive different fire regimes in a given environment [38,39] but are unlikely to drive biogeographical differences.

One of the limitations of our study is the use of a biased sampling of the species distribution (from GBIF records). However, we have minimized the possible sampling bias by (i) excluding GBIF records that were closer than 1 km, (ii) selecting 500 randomly selected plots (subsetting) for the statistics (e.g. [15]), and (iii) considering the spatial pattern in analyses (SAR models, table 2). The observed patterns are strong and consistent in two pairs of species, suggesting that the potential sampling bias is unlikely to affect the conclusions. We have analysed the occurrence of surface versus crown fire; however, there is a significant variability in the fire regime within each of these types (e.g. in frequency and intensity), which also deserves analysis. Small-scale variability in climate, topography and fuel distribution is likely to be responsible for such variability [6,40–42]. Human influences occurring at small spatial scales could also override environmental controls (e.g. [43]).

While surface and crown fires may be the dominant fire regimes characterizing each continent (boreal Eurasia versus boreal America, respectively), both continents are large and diverse and therefore environmentally heterogeneous. As such, there is evidence of crown fires in some areas of Eurasian boreal forests [44] and surface fires in some areas of American boreal forests [45]. In addition, although serotiny is absent in Eurasian forests, North American boreal forests also host non-serotinous trees, including species of pine (e.g. *Pinus resinosa*, a thick-barked tree with self-pruning, living under surface fire regimes [45]) and spruce (*Picea glauca*, a fire-sensitive tree that colonizes post-fire if the recurrence is not too high) [46]. Local factors (e.g. sandy soils in *Pinus resinosa* habitats) are important for driving fire regimes at finer scales, allowing the persistence of these other species in pockets on these continents. This is why we focused on species distributions, i.e. subcontinental variability, to explain the difference in plant traits between continents. By focusing on congeneric *Picea* and *Pinus* that are widespread, inhabit different continents, and have different traits (serotinous/non-serotinous in North America/Eurasia), we could restrict our analysis to the species and geographies that are relevant to our question.

We conclude that, at broad scales, the distribution of surface and crown fires across the boreal forest is controlled by top-down (environmental) factors and maintained by eco-evolutionary dynamics.

## Data Availability

Supplementary material is available online at [47].
